# Mechanism and regulation of the nonsense-mediated decay pathway

**DOI:** 10.1093/nar/gkw010

**Published:** 2016-01-14

**Authors:** Nele Hug, Dasa Longman, Javier F. Cáceres

**Affiliations:** Medical Research Council Human Genetics Unit, Institute of Genetics and Molecular Medicine, University of Edinburgh, Western General Hospital, Edinburgh, EH4 2XU, UK

## Abstract

The Nonsense-mediated mRNA decay (NMD) pathway selectively degrades mRNAs harboring premature termination codons (PTCs) but also regulates the abundance of a large number of cellular RNAs. The central role of NMD in the control of gene expression requires the existence of buffering mechanisms that tightly regulate the magnitude of this pathway. Here, we will focus on the mechanism of NMD with an emphasis on the role of RNA helicases in the transition from NMD complexes that recognize a PTC to those that promote mRNA decay. We will also review recent strategies aimed at uncovering novel trans-acting factors and their functional role in the NMD pathway. Finally, we will describe recent progress in the study of the physiological role of the NMD response.

## INTRODUCTION

Cells have evolved different surveillance mechanisms to target messenger RNAs (mRNAs) with mutations that would otherwise lead to errors in the synthesis of proteins, and also to eliminate other incorrectly processed cellular RNAs. These mechanisms operate both in the cell nucleus and cytoplasm (1). One of the best-studied RNA surveillance pathways is the Nonsense-mediated decay (NMD) pathway, which targets mRNAs harboring premature termination codons (PTC) for degradation. This mechanism operates in the cytoplasm and is intimately linked to translation termination (2,3).

**Figure 1.**
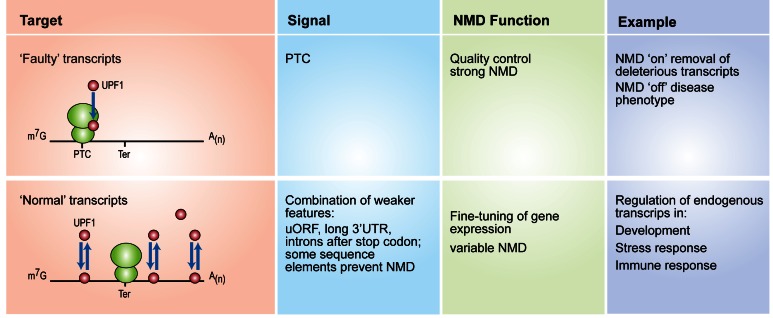
Dual role of the Nonsense-mediated decay (NMD) pathway. NMD degrades PTC-containing transcripts (‘faulty’ transcripts) (top panel) and also regulates the expression of naturally occurring transcripts (‘normal transcripts’), acting as a fine-tuning mechanism of gene expression (bottom panel). Green spheres represent both ribosomal subunits, whereas red circles depict the core NMD factor, UPF1.

Initially, NMD was described as a post-transcriptional mRNA quality control mechanism responsible for the removal of PTC-containing mRNAs, which if left intact, would lead to production of truncated proteins with predicted deleterious effects for the organism. From a medical perspective, this suggests that the NMD pathway has a role in the modulation of the phenotypic outcome of genetic disorders that are caused by the presence of a PTC (4,5). However, it has become evident in recent years that this pathway is not solely dedicated to the destruction of PTC-containing transcripts, but that it also has an important role in controlling the expression of naturally occurring transcripts (6–8) (Figure 1). This general role of the NMD pathway on gene expression requires the existence of buffering mechanisms to tightly regulate the magnitude of the NMD response upon environmental and/or genetic insults. Accordingly, a negative feedback regulatory network that controls the levels of core NMD factors operates in mammals (9,10), in nematodes and zebrafish (11), and also in plants (12). Interestingly, the magnitude of the NMD response has also been shown to be variable among individuals (13). The NMD pathway is not exclusively dedicated to mRNAs, as shown by the substantial number of long non-coding RNAs (lncRNAs) that are substrates of NMD in *Arabidopsis*, *S. cerevisiae* and mouse ES cells (14–16). This is not entirely surprising, taking into account the recently revealed association of the translation machinery with lncRNAs (17).

In this article, we will cover recent advances regarding the NMD mechanism, building on the roles of core NMD factors, the functions of RNA helicases in NMD complex assembly, as well as recent strategies used to identify novel NMD factors. Finally, we will review the crucial role of the NMD mechanism in buffering gene expression and its impact on cell physiology.

### PTC definition

A crucial aspect of the NMD pathway is the ability to distinguish normal termination codons from PTCs. Despite the relatively high conservation of NMD core factors across evolution, the mechanism of PTC definition varies across different species. In mammals, NMD is intimately linked to pre-mRNA splicing, and mRNAs harboring a PTC 50 to 55 nucleotides upstream of the final exon–exon junction are efficiently degraded (18–21). This is signaled by the presence of the exon junction complex (EJC), a multi-subunit protein complex, which is deposited 20 to 24 nucleotides upstream of an exon–exon junction during pre-mRNA splicing (22,23). The EJC remains associated to the mRNA until it is displaced by the translation machinery with the help of the ribosome-associated protein PYM acting as an EJC disassembly factor (24,25,26). Recent transcriptome-wide analysis of EJC deposition established that EJCs are not equally assembled at every exon junction, as previously hypothesized (27,28); indeed, approximately half of all EJCs are present at non-canonical positions (29). A remaining challenge is to understand whether the observed variation on EJC loading affects NMD efficiency. By contrast, PTC definition occurs independently of exon boundaries in *S. cerevisiae*, where the distance between the PTC and the 3′ end defines NMD, as proposed by the faux 3′ UTR model (30,31). In *Schizosaccharomyces pombe* splicing enhances NMD; however, EJC components are not required for NMD and what seems to enhance NMD is the proximity of the intron to the PTC (32). The presence of introns is also not required to define PTCs in *Drosophila* or in *C. elegans*, exposing a significant level of mechanistic diversity in this critical step of the NMD process (33,34).

### NMD mechanism and the role of RNA helicases in NMD progression

The initial identification of factors with roles in the NMD pathway was achieved by means of unbiased genetic screens in *Caenorhabditis elegans* and *Saccharomyces cerevisiae*. This led to the identification of seven genes in nematodes, termed *smg-1–7* (suppressor with morphological effect on genitalia), given that mutations of these genes led to abnormal morphogenesis of the male bursa and the hermaphrodite vulva (35,36). Importantly, *smg* mutant worms are viable, indicating that NMD is not essential in nematodes. Similarly, three genes, termed *UPF1–3* (for up-frameshift), that are orthologues of *C. elegans smg-2, smg-3* and *smg-4* genes, were identified in *S. cerevisiae* (37,38). Homology searches led to the identification of orthologous genes in other species, including *Arabidopsis, Drosophila* and mammals (39).

**Figure 2.**
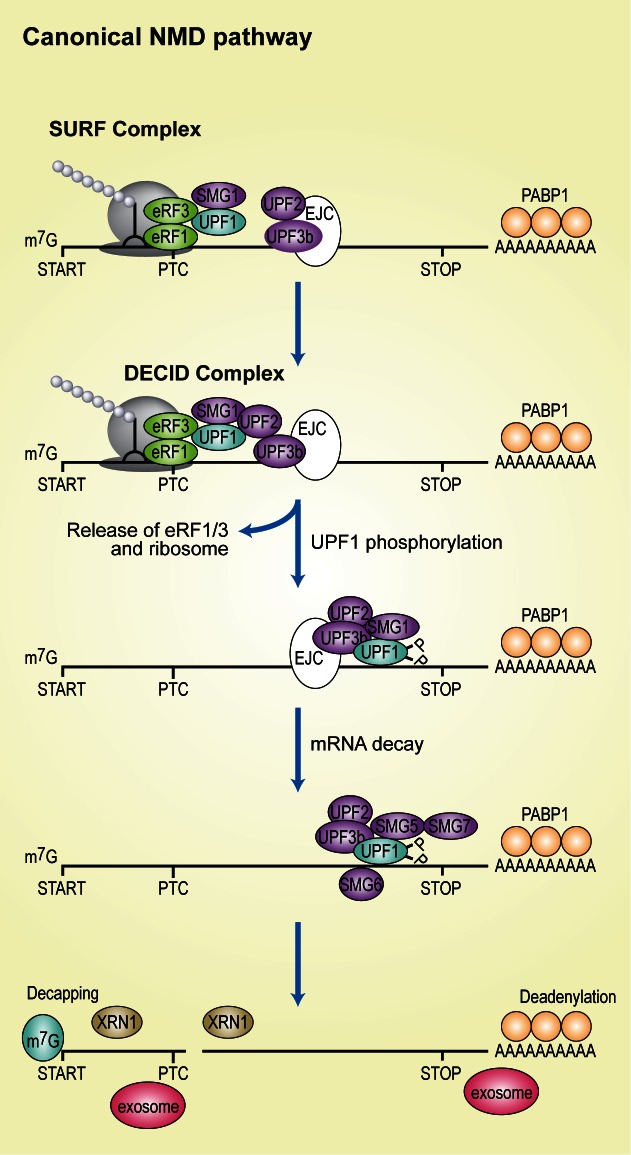
Mechanism of NMD activation in mammalian cells, indicating the transition from the surveillance complex (SURF) to the decay-inducing complex (DECID). UPF1 and its associated kinase, SMG1, bind to the eukaryotic release factors eRF1 and eRF3 to form the SURF complex in the vicinity of a premature termination codon (PTC). A subsequent interaction of this complex with UPF2, UPF3b and an exon junction complex (EJC) downstream of the PTC triggers the formation of the DECID complex, resulting in UPF1 phosphorylation and release of eRF1 and eRF3. Phosphorylated UPF1 acts to recruit SMG5, SMG6 and SMG7, and general mRNA degradation factors that lead to mRNA degradation.

RNA helicases have a central role in the mechanism of NMD progression. In general, these enzymes can use adenosine triphosphate (ATP) to translocate along nucleic acids, potentially unwinding secondary structure and acting to remodel RNA-protein complexes. Alternatively, they might act as ‘‘place markers’’ remaining temporarily fixed in a defined position while signaling to, or directly recruiting, the degradation machinery (40,41). In the latter case, RNA helicases clamp the RNA in an ATP-dependent fashion to provide nucleation centers to assemble larger RNA-protein complexes. The central component of the NMD pathway in all organisms studied is the protein UPF1/SMG2, an ATP-dependent RNA helicase of the SF1 superfamily, which undergoes cycles of phosphorylation and dephosphorylation that are essential for NMD progression. Phosphorylation of UPF1 is carried out by the SMG1c complex, comprised of the protein kinase SMG1, a phosphoinositide 3-kinase (PI3K)-like kinase and two additional subunits, SMG8 and SMG9 (42–44). Initially, UPF1 associates with SMG1 and acts as a clamp, interacting directly with the eukaryotic release factors eRF1 and eRF3 to form the so-called surveillance complex (SURF) in the vicinity of the PTC (Figure 2). Two subunits of the SMG1c complex, SMG8 and the NTPase SMG9 associate tightly with SMG1 and regulate its activity through the induction of conformational changes, with SMG8 binding to the preformed SMG9-SMG1 complex and maintaining the kinase in its inactive state (45–47). Subsequently, the SURF complex interacts with UPF2, UPF3b and an EJC downstream of the PTC to form the decay-inducing complex (DECID) that triggers UPF1 phosphorylation and dissociation of eRF1 and eRF3 (48–50) (Figure 2). As a consequence of the remodeling of NMD complexes, UPF1 adopts its active helicase confirmation due to the reorganization of its inhibitory domains through association with UPF2 (51–54). The active UPF1 helicase functions as RNPase translocating along the mRNA, resolving secondary structure and clearing the mRNA from proteins allowing access to nucleases (55,56). The activated NMD complex consisting of UPF1, UPF2 and UPF3b is translocated from its position upstream of the EJC toward the 3′ of the EJC (57) (Figure 2). Subsequently, phosphorylated UPF1 associates with the phospho-binding proteins SMG5, SMG6 and SMG7, and general mRNA degradation factors and further rearrangements of this complex lead to mRNA degradation (58). SMG6 itself is an endonuclease, which can form both phospho-dependent and phospho-independent interactions with UPF1(59–62). SMG6 cleaves NMD targets in the vicinity of the PTC (59,63), leading to the initiation of NMD-mediated RNA degradation (64–66). SMG5 and SMG7 bind as a heterodimer to phosphorylated UPF1. The SMG7 subunit recruits directly POP2, the catalytic subunit of the Deadenylase complex (67) and additionally initiates decapping and XRN1-catalyzed 5′-3′ degradation (68). This canonical mammalian NMD pathway is not universal, since alternative NMD branches that are independent of UPF2, UPF3b or the EJC have been described (69–71).

**Figure 3.**
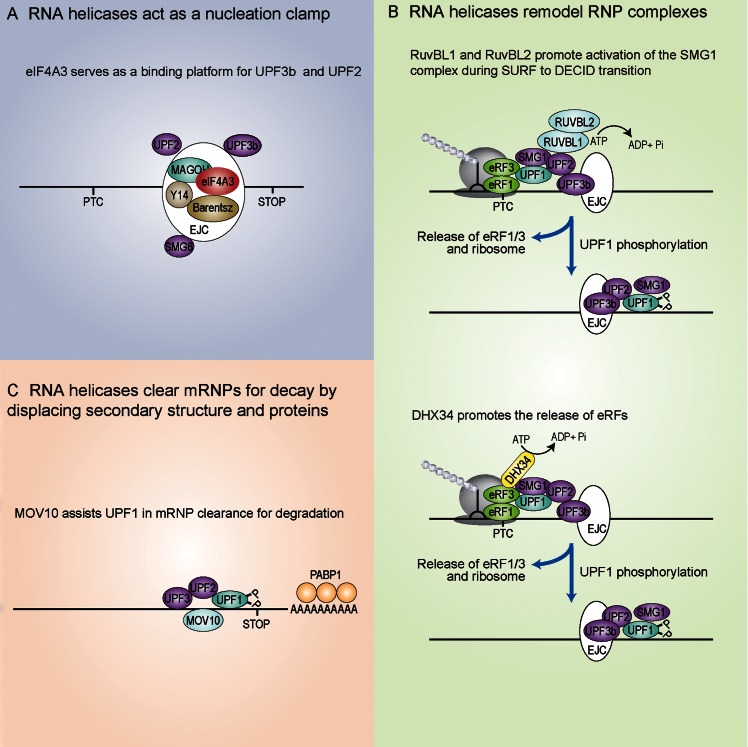
Role of RNA helicases in NMD. (**A**) The exon junction complex (EJC) component, eIF4AIII, nucleates NMD factors, promoting interactions with UPF2 and UPF3b. (**B**) RuvBL proteins promote activation of the SMG1 kinase during initial stages of NMD (upper panel). DHX34 promotes the transition from the SURF to DECID complex (lower panel). (**C**) Function of the RNA helicases UPF1 and MOV10 in mRNP clearance.

Another RNA helicase with a role in EJC-mediated NMD progression is eIF4AIII, a core component of the EJC, that binds RNA in an ATP-dependent fashion and recruits additional EJC factors, namely the heterodimer Y14/MAGOH and Barentz (BTZ, MLN51 or CACSC3)(72,73) (Figure 3A). The crystal structure analysis of this complex revealed that eIF4AIII binds RNA in an ATP-dependent fashion and recruits the other EJC core factors (72,73). The heterodimer Y14/MAGOH stabilizes the high affinity of eIF4AIII for RNA by preventing the ATP hydrolysis of eIF4AIII and further primes it to bind to Barentz (74,75). This arrangement allows eIF4AIII to clamp several proteins onto RNA in a stable and sequence-independent manner (76), which in turn is used by the NMD machinery to recognize an aberrant PTC (Figure 3A). Thereby, a short motif of the core NMD factor UPF3b binds to the EJC core factors and serves as a platform to assemble an active NMD complex (52,77). Two other DEAD box proteins DDX5 and DDX17 have also recently been described to interact with UPF3b and this interaction seems crucial for the degradation of a limited subset of NMD substrates (78).

Additional RNA helicases also function as auxiliary factors in the NMD pathway. Through a process involving their ATPase activities, the RNA helicases RUVBL1 and RUVBL2 associate with the SURF complex and promote the transition to the DECID complex (Figure 3B, upper panel) (79). Given their tight association to the SMG1 kinase, it can be speculated that RUVBL1, 2 contribute to the regulation of the SMG1–8–9 complex by promoting the activation of the large SMG1 kinase molecule. It was recently shown that the RNA helicase DHX34, a member of the DExH/D box family of proteins, associates with the SURF complex and promotes the transition to the DECID complex (80). Whereas members of this family have been originally described as RNA helicases that unwind RNA, it has also been found that other family members function as RNPases that remodel RNA-protein interactions (81,82). Thereby, DHX34 probably affects the remodeling of the SURF complex by promoting the dissociation of the eukaryotic release factors eRF1 and eRF3 in an ATP-dependent manner from the SURF complex at the PTC (80) (Figure 3B, lower panel). It was recently shown that another UPF1-like RNA helicase, MOV10, preferentially binds to 3′ UTR regions, as is the case with UPF1 (83,84), and contributes to degradation of UPF1-regulated transcripts (85,86) (Figure 3C). Whether MOV10 helicase acts in a redundant way with the UPF1 helicase as an RNA clearance factor to resolve secondary structures and displace proteins or if the two proteins perform distinct actions remains to be clarified.

### Non-EJC dependent models of NMD

Although it has been clearly established that the presence of an EJC downstream of a PTC promotes NMD in mammalian cells, there is also increasing evidence of an active NMD response in its absence. Therefore, NMD activation can rely on both EJC-dependent and EJC-independent pathways (87). Alternative models for NMD activation that do not require the presence of an EJC have been described in other organisms, such as yeast and nematodes, but could also operate in human cells (88). In particular, mRNAs harboring long 3′ UTRs have been shown to be sensitive to NMD, irrespective of the presence of an EJC (83). A central issue in NMD concerns the mechanism by which UPF1 is recruited to an NMD target. Recent studies showed that UPF1 binds target RNAs before mRNA translation and subsequently translating ribosomes displace it from coding sequences leading to the accumulation of UPF1 at 3′ UTRs (86,89). This observation challenges the assumption that UPF1 recruitment marks mRNA for degradation. Moreover, UPF1 binding is not enriched on endogenous transcripts that are upregulated in the absence of UPF1 (89,90). A recent study proposed that binding of phosphorylated UPF1 (P-UPF1) marks mRNAs for degradation. It was shown that P-UPF1 is enriched on endogenous transcripts degraded by NMD and predominantly unphosphorylated UPF1 is released from non-targeted transcripts in an ATP-dependent manner (91). However, P-UPF1 binding is not exclusive to NMD targets, and it is also not apparent what makes UPF1 ‘stick’ preferentially to NMD targets and get phosphorylated. Further clues about how UPF1 discriminates NMD targets come from a recently published RIP-seq data of wild-type or ATPase-deficient UPF1 cells, which showed that UPF1 release from non-target mRNAs rather than UPF1 binding itself was more important for NMD target selection. Faster dissociation of UPF1 from non-target mRNAs requires correct ATPase activity of UPF1 and its dissociation is enhanced by translation and PABC1 binding, also in an ATPase-dependent manner (92). Prematurely terminated translation associated with a PTC leads to mRNA degradation and it has been proposed that this is due to a competition between UPF1 and the poly (A) binding protein, PABPC1, for binding to the translation release factor, eRF3 (93). As such, the distance from the PTC to the poly (A) tail is a determining factor on whether UPF1 will bind near the PTC (30,71,94,95).

### How are NMD targets recognized?

Even though our understanding of the NMD process is rapidly increasing, the important question of how NMD targets are selected in a global scale still remains unresolved. So what rules govern NMD target selection? Although both EJC-dependent or independent models for target recognition can be applicable to a selected number of targets, the situation is less clear when analyzing high-throughput NMD targets. For instance, profiling of transcripts regulated by UPF1 revealed that many transcripts that are upregulated in the absence of UPF1 lack recognizable NMD features, whereas many transcripts that would be predicted to be NMD targets are unaffected by UPF1 depletion (96–98).

The emerging picture suggests that likely no single NMD feature will be globally sufficient to elicit NMD. Instead, a combination of NMD-targeting and NMD-antagonizing features contributes to determine NMD susceptibility of any given mRNA. For some mRNAs, a canonical model will apply, where a PTC situated 50–55 nucleotides upstream of an EJC elicits robust mRNA downregulation (99). This is certainly the case for ‘faulty’ PTC-containing transcripts for which NMD was originally described (see Figure 1). In cells, these transcripts are usually rare, and only produced as a result of genomic mutation or incorrect RNA processing, and are often associated with diseases. They are very efficiently degraded by NMD, thus preventing the production of truncated proteins. For other mRNAs, a distinct combination of NMD features will determine if, and how efficiently, transcripts are degraded. This mode of target determination is likely used for a vast majority of endogenous NMD targets, where NMD is an essential tool for the fine-tuning of gene expression.

### Additional NMD trans-acting factors and their role in the regulation of NMD

**Figure 4.**
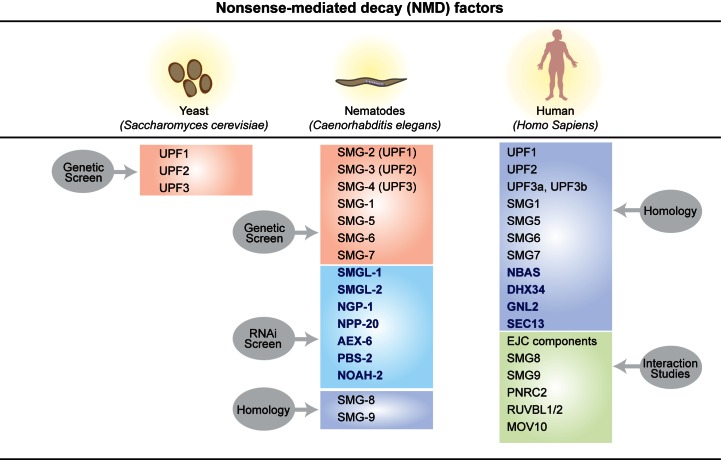
NMD factors in yeast, nematodes and humans, indicating the method by which they were first identified.

A variety of strategies have been used in an attempt to uncover novel factors with a role in the NMD response, including forward and reverse genetic screens and interactome studies (Figure 4). A limitation of forward genetic screens lays in their inability to identify genes that are essential for viability. Additionally, the search of novel NMD factors using mutagenesis in *C. elegans* was somewhat undermined by the fact that almost 90% of *smg* mutations identified were alleles of *smg-1, smg-2* or *smg-5* (35,36,100). This limitation was overcome in reverse genetic screens using RNAi that allows the identification of NMD factors independently of their abundance or whether they are essential for cell survival.

In this context, the Interactome-Mass spectrometry approach emerges as a promising avenue for the identification of abundant proteins that form part of NMD complexes. A study using a yeast two-hybrid approach with UPF1 as bait identified human proline-rich nuclear receptor coregulatory protein 2 (PNRC2), as an interactor. This factor was also found to interact with the decapping activator, DCP1a, which led to a model whereby phosphorylated UPF1 acts as a platform to recruit PNRC2, providing a link with mRNA degradation (101,102). A comprehensive search using stable isotope labeling by amino acids in cell culture (SILAC) was designed to distinguish between proteins binding to either the hypo- or hyper-phosphorylated form of UPF1. This resulted in the identification of several RNA-binding proteins that preferentially associate with hyper-phosphorylated UPF1 in the nucleus. It also confirmed the eukaryotic translation initiation factor 3 (eIF3) as a UPF1 interactor (103). An interactome search for the RNA helicase MOV10, which belongs to the UPF1-like group of the helicase superfamily 1 (SF1)(104), identified UPF1as the major interactor (86). Finally, use of SMG1 as bait identified RuVB-like 1 (RUVBL1) and RuVB-like 2 (RUVBL2) proteins, two adenosine triphosphatases that are part of (AAA+) family of proteins (Figures 3 and 4). These proteins have been reported to be involved in several cellular functions, such as transcription, DNA repair, telomere maintenance and RNA modification, and also shown to act in the initial stages of the NMD pathway. Other SMG1 interactors identified in the same experiment were RPB5, a subunit of RNA polymerases I, II and III (105), as well as the homolog of a nematode NMD factor, termed SMG10, both of which also form part of the RUVBL1/2 complex (79).

Use of a GFP-reporter-based RNAi screen in *C. elegans* resulted in the identification of two novel NMD factors, termed *smgl-1 and smgl-2*, that unlike *smg* genes are essential for viability, strongly suggesting that these two genes have roles in NMD but also fulfill other essential functions in nematodes (33) (Figure 4). Both genes are conserved throughout evolution with clear orthologues in mouse, human and fugu. The *C. elegans* gene *smgl-1* corresponds to human NBAS (Neuroblastoma amplified sequence), also known as NAG (for Neuroblastoma amplified gene). The *C. elegans smgl-2* gene corresponds to human DHX34 (DEAH box protein 34), a DExH/D box protein (Figure 3B, lower panel). Both NBAS and DHX34 are *bona fide* NMD factors both in human cells, as well as in zebrafish (33,106). Identification of target genes regulated by DHX34 and NBAS, in human, zebrafish and also in *C. elegans* revealed a large co-regulation of targets between DHX34, NBAS and the core NMD factor UPF1 in all species (11). The *NBAS* gene was initially identified as a gene amplified in human neuroblastomas (107,108), and later found to encode a peripheral membrane protein that is a component of the Syntaxin 18 complex, with a role in Golgi-to-endoplasmic reticulum retrograde transport (109). Interestingly, mutations in *NBAS* have been found in several human conditions, including a hereditary short stature syndrome in the Siberian Yakut population, characterized by optic nerve atrophy and Pelger–Huët anomaly of granulocytes (SOPH syndrome)(110), as well as in patients with a multisystem disease involving liver, eye, immune system, connective tissue and bone (111). Another recent report identified compound heterozygous *NBAS* mutations with recurrent acute liver failure in infancy in a group of patients of European descent (112). It remains to be seen whether the phenotypes of NBAS mutants are due to compromised NMD response and/or defects in retrograde transport between the ER and Golgi. Currently it is unclear, how NBAS contributes to NMD in mechanistic terms; however, we have recently found that NBAS interacts with the core NMD factor UPF1 (Longman and Caceres, unpublished data). Combined with a reported role in ER-Golgi vesicular trafficking (109), we speculate that NBAS might represent a connecting link between NMD and transcripts entering the secretory pathway. Interestingly, profiling of transcripts regulated by NBAS showed significant enrichment for genes involved in the cellular stress response (11).

The original NMD RNAi screen in *C. elegans* was revisited; using a novel RNAi library that included many previously untested genes (113,114). This new screen resulted in the identification of five novel nematode NMD genes that are highly conserved throughout evolution and are required for NMD in nematodes (Figure 4) (115). Two of their human homologs, *GNL2* (*ngp-1*) and *SEC13* (*npp-20*), were also found to act in the NMD pathway in human cells (115). Moreover, expression profiling showed that a significant proportion of transcripts that are regulated by GNL2 and SEC13 were also largely upregulated when canonical NMD factors UPF1 and UPF2 were depleted (Casadio, Longman and Caceres, unpublished). The *GNL2* gene encodes a putative GTPase, whose yeast homolog, Nog2p, is involved in the processing of the pre-60S ribosomal particles (116), whereas human SEC13 is a constituent of the endoplasmic reticulum and the nuclear pore complex (NPC) (117). Whether these previously described roles of GNL2 and SEC13 are related to their role in NMD will require additional investigation. This screen also identified the *C. elegans* gene, *noah-2*, which is present in *Drosophila melanogaster* (*nompA*) but absent in humans, and has a role as an NMD factor in fruitflies (115). Interestingly, the expression of *nompA* is restricted to the type I sensory organs of the peripheral nervous system (PNS), suggesting the possibility that NMD can act in a tissue-specific manner.

### Physiological role of the NMD response

The NMD response has been shown to be variable among different cell-types and tissues (118,119), and even among individuals where this can be correlated with clinical presentations of human diseases caused by the presence of a PTC (13). What are the mechanisms that lead to a variable NMD response? One such mechanism could be the variation in the relative abundance of RNPS1, a peripheral component of the EJC, observed in different HeLa cells subtypes that correlates well with the magnitude of the NMD response (120). The relatively recent realization that NMD acts to regulate endogenous gene expression of mRNAs lacking a PTC strongly suggested that the NMD response itself has to be precisely regulated in order to avoid undesirable alterations to the gene expression program of cells and tissues. One way to regulate the NMD activity is via a negative feedback regulatory network whereby a large proportion of core NMD factors are negatively regulated by NMD. This feedback loop was initially observed in mammalian cells, and was later also found in nematodes, zebrafish and plants (118).

The NMD pathway is not essential in nematodes where mutations in the core NMD factors (*smg1–7*) lead to discrete phenotypes (35). A similar scenario is found in yeast, where the loss of the Upf proteins has no obvious effect on growth (37). By contrast, targeted disruption of mammalian core NMD factors results in embryonic lethality in the mouse, as observed for *Upf1* (121), Upf2 (98) or *Smg6* (122). Along the same lines, *Smg1* is required for embryogenesis and was shown to regulate target genes via alternative splicing coupled to NMD (123). The phenotypes observed with inactivation of mouse core NMD genes could be interpreted as indicating an essential role for the NMD pathway in mammals. An alternative explanation is related to the existence of additional cellular functions for UPF proteins (124). These include a reported role for UPF1 in genome stability in human cells (125), as well as its role in the SMD pathway (Staufen-mediated decay), whereby UPF1 is recruited to the 3′ UTRs of mRNAs bound by the RNA-binding protein STAUFEN 1 and induces mRNA degradation (126,127). UPF1 has also been shown to be involved in regulating the degradation of histone mRNAs, where it is recruited by the stem-loop binding protein (128). The *Smg1* gene has also an additional role in genotoxic stress (129). As in the mouse system, Drosophila Upf1 and Upf2 are also required for animal development and viability (130). By contrast, Drosophila Upf3 plays a peripheral role in the degradation of most NMD targets and is not required for development or viability (131). The role of the NMD response and/or of individual NMD factors during development and also during neuronal development has recently been reviewed (93,119). Below, we will focus on a few examples on how the NMD pathway impacts on several physiological processes, including the stress response, the immune response and on viral replication.

### Stress response

In response to stress, cells initiate a complex cascade of events leading to dynamic changes in gene expression that are designed to alleviate stress and restore homeostasis, or trigger apoptosis. It was observed that NMD is repressed by a variety of stress conditions, such as hypoxia, nutrient deprivation or infection (132,133). This inhibition is, at least partially, mediated by phosphorylation of the translation initiation factor eIF2α, which leads to inhibition of mRNA translation and is a common step for the initiation of many stress pathways (134). This results in many transcripts that are normally degraded by NMD being upregulated, including those encoding factors that are required during stress response, helping to establish a more robust stress response and increasing cell survival (135). NMD also modulates the unfolded protein response (UPR), which is triggered by endoplasmic reticulum (ER) stress. A chronic activation of the UPR contributes to the pathogenesis of a wide variety of human disorders; thus, UPR activation must be tightly regulated. It was shown that NMD directly targets mRNAs encoding several essential UPR components, including many UPR sensors (136–138). In this way, NMD controls the threshold of UPR activation, and prevents the inappropriate response to harmless levels of stress (136). Additionally, NMD inhibition itself, can also induce UPR (137,138). It is possible that inhibition of NMD overloads the ER with truncated misfolded proteins that themselves could generate sufficient stress signal. However, the extent of this NMD-mediated effect on UPR activation remains to be determined. Moreover, NMD inhibition leads to the activation of autophagy that decreases accumulation of detrimental proteins in the cell. Autophagy activation is partially due to the stabilization of the NMD target ATF4 mRNA (139). Conversely, hyperactivation of the NMD response blocks the induction of autophagy in response to cellular stresses. In summary, under normal conditions, NMD activity is required for protecting cells from inappropriate activation of UPR by innocuous stimuli. In response to stress, NMD inhibition augments UPR response by increased expression of transcripts that encode UPR sensors such as IRE1α. NMD re-activation then helps terminate the stress response and restore homeostasis. Therefore, NMD helps to fine-tune the threshold of UPR response, and the activity of NMD is in turn regulated by the UPR.

### Immune response

A robust immune response is needed for preventing or limiting infection; however, if left uncontrolled it can lead to pathologies or death. It is therefore crucial that organisms are able to maintain immune homeostasis by suppressing or switching off the immune response. It was recently proposed that NMD plays an active role in the regulation of the immune response. Cytokines are signaling molecules (e.g. IL-6 or TNF) that modulate the inflammatory immune response. In response to infection, cytokines are rapidly upregulated and initiate an immune response cascade via binding to their receptors at the surface of the cells. It was recently shown that UPF1, together with the RNA-binding protein, Regnase-1, regulates the early (acute) phase of inflammation response by degrading cytokine mRNAs. Regnase-1 binds to a stem-loop in 3′ UTRs of translationally active cytokine mRNAs and acts together with UPF1 to downregulate these targets, whereas the role of another RNA-binding protein Roquin, that controls the late (chronic) phase of inflammation, is independent of UPF1 (140). Although it is not entirely clear that the action of Regnase-1 together with UPF1 represents NMD per se, the helicase activity of UPF1 is required. Another way that NMD could affect the inflammation response is via controlling the stability of the cytokine receptor mRNAs. One recently documented example is the complex regulation of the human CCR5 cytokine receptor mRNA stability (141). CCR5 mRNA harbors a programmed −1 ribosomal frameshift (−1PRF) signal directed by an mRNA pseudoknot that is formed by mRNA–miRNA interactions. This −1PRF directs the translating ribosome to a PTC, leading to mRNA downregulation.

A better understanding of NMD modulation during immune response can be useful for the development of new therapies for autoimmune diseases or cancer. For example, upregulation of NMD during chronic inflammation can help to restore immune homeostasis. On the other hand, blocking NMD in cancer may result in the synthesis of tumor-specific proteins that can increase natural immune response directed against the tumor (142). This could indeed be a feasible approach, as it was recently demonstrated that the increase of intracellular calcium by commonly used cardiac glycosides inhibits NMD (143). A role for NMD in the control of the immune response is also seen in plants, where this pathway contributes to innate immunity in *Arabidopsis* (144–146). NMD acts to downregulate numerous TIR domain-containing, nucleotide-binding, leucine-rich repeat (TNL) immune receptor-encoding mRNAs. Bacterial infection of plants leads to host-programmed inhibition of NMD, resulting in the upregulation of those naturally NMD-regulated TNL transcripts. By contrast, constitutive NMD activity prevents accumulation of TNL receptors and impairs plant defense. Thus, a mechanism of host-regulated NMD contributes to disease resistance by controlling the threshold for activation of TNL resistance pathways (145).

### Viral replication

Apart from its role in immune response regulation, NMD also serves as a natural barrier to virus replication. A genome-wide RNAi screen in HeLa cells looking for host factors that restrict virus replication identified several components of the NMD pathway (147). Downregulation of UPF1, SMG5 and SMG7 led to an increase in the level of viral proteins and higher viral infection. Viruses have also evolved mechanisms to evade NMD-mediated degradation. For example, Rous sarcoma virus harbors a stability element in its 3′ UTR (RSE, for Rous sarcoma virus stability element) that protects the viral RNA from NMD. It was hypothesized that this viral stability element may prevent UPF1 function (148,149). The HTLV-1 virus Tax and Rex proteins also inhibit NMD (150,151); however, their mode of action is not entirely clear. It has been shown that the Tax protein interacts with UPF1 and with a component of eIF3, INT6 that also has a proposed role in NMD. Tax increases the accumulation of phosphorylated UPF1 in P- bodies and this leads to enhanced stability of HTLV-1 mRNAs (150). Similarly, plants also employ NMD to restrict viral replication by destabilizing viral transcripts containing internal stop codons or long 3′ UTRs. As expected, plant viruses also evolved mechanisms that either evade NMD, or modify host endogenous NMD activity (152). Therefore, the host NMD response that reduces viral infection and is in turn counteracted by viruses modulating NMD seems to be an evolutionarily conserved relationship.

### NMD inhibitors and therapeutic possibilities

NMD activity can be modulated by many cellular perturbations. Treatment of human cells with chemotherapeutic compounds results in a pronounced decline in NMD activity. This is partly due to the proteolytic production of a dominant-negative form of the key NMD factor UPF1 and results in the upregulation of genes involved in the response to apoptotic stresses, leading to cell death (153). Furthermore, caspases cleave both UPF1 and UPF2 during apoptosis, impairing the NMD response (154). In an interesting link with a human disease, it has been recently reported that increased expression of the double homeobox transcription factor DUX4, which is observed in patients with the muscular dystrophy, Facioscapulohumeral muscular dystrophy (FSHD), triggers proteolytic degradation of UPF1, leading to pronounced NMD inhibition. DUX4 mRNA is itself an NMD target, thus, inhibition of NMD by DUX4 protein stabilizes DUX4 mRNA through a double-negative feedback loop in FSHD muscle cells (155).

The development of inhibitors of NMD could be important to gain mechanistic insights into NMD function but could also prove to be important for therapeutic purposes. The search for NMD inhibitors is currently approached by means of several strategies. A screen in HeLa cells stably expressing an NMD reporter that used a library of clinically licensed compounds identified 5-azacytidine, an analog of the naturally occurring nucleoside cytidine, which has been previously approved for the treatment of myelodysplastic syndrome and myeloid leukemia (156). This inhibitory effect of 5-azacytidine on NMD, depends on the induction of MYC expression, which was previously shown to inhibit NMD activity (157). A small molecule inhibitor of NMD, termed NNMDI 1, was shown to stabilize hyperphosphorylated isoforms of UPF1 and to compromise its interaction with SMG5 (158). Compounds that disrupt the SMG7-UPF1 complex and inhibit NMD have also been recently identified. These compounds when combined with a PTC ‘read-through’ drug led to restoration of full-length p53 protein in cells harboring a PTC-mutated p53 (159). Pateamine A (PatA), a natural product first isolated from marine sponges, was also shown to inhibit NMD through a direct interaction with the EJC component, eIF4AIII. Importantly, this PatA-mediated inhibition of NMD is independent of the previously reported role of this compound in inhibition of translation initiation (160). The dietary compound curcumin has also been shown to inhibit the NMD pathway by downregulating the expression of core NMD factors at the transcriptional level (161). Finally, pyrimidine derivatives have been identified as hSMG-1 kinase inhibitors (162). In many cases, expression of a PTC-containing mRNA can be advantageous for the cell, when the production of a truncated protein is less harmful than elimination of its encoding mRNA by NMD. This is particularly important in the case of certain human diseases caused by mutations that introduce PTCs. A number of drugs have been identified that induce suppression of translation termination at PTCs in mammalian cells (163,164). This PTC suppression therapy, which is currently in clinical trials for treatment of several genetic diseases caused by PTCs, can be combined with strategies to inhibit NMD. One such strategy of NMD inhibition combined with PTC read-through was recently reported (165). Here, antisense oligonucleotides (ASOs) designed to block assembly of an EJC downstream of PTCs and inhibit NMD in a gene-specific fashion were combined with read-through compounds. This approach restored expression of full length protein from a nonsense-mutant allele (165). Thus, these complementary strategies could have an important role in alleviating the phenotypic consequences of a wide range of genetic diseases caused by the presence of a PTC.

## CONCLUSION

In the last few years, there has been a significant increase in our understanding on how the NMD mechanism operates, how the different sub-complexes are assembled and the role of some of the NMD trans-acting factors. The realization of a more general role for NMD in regulating gene expression raised some new questions, such as how is the NMD response buffered, how are non-PTC-containing endogenous transcripts targeted by the NMD machinery and whether there are more trans-acting factors yet to be identified. A full understanding of the role of the NMD response in the physiology of cells represents both an interesting mechanistic challenge but also an opportunity for future therapeutic interventions.
